# Maturation‐Dependent Constraints in the Ability of Human Monocytes to Initiate Trained Immunity

**DOI:** 10.1002/mco2.70939

**Published:** 2026-10-04

**Authors:** Michael Eigenschink, Elsa Davogg, Manuel Pristner, David Seki, Marcus Arzmüller, Herbert Kiss, Alex Farr, Angelika Berger, David Berry, Benedikt Warth, Lukas Wisgrill

**Affiliations:** ^1^ Department of Pediatrics Division of Neonatology Pediatric Intensive Care and Neuropediatrics Medical University of Vienna Vienna Austria; ^2^ Comprehensive Center for Pediatrics Medical University of Vienna Vienna Austria; ^3^ Department of Food Chemistry and Toxicology University of Vienna Vienna Austria; ^4^ Joint Microbiome Facility of the Medical University of Vienna and the University of Vienna Vienna Austria; ^5^ Department of Microbiology and Ecosystem Science Centre For Microbiology and Environmental Systems Science University of Vienna Vienna Austria; ^6^ Department of Obstetrics and Gynecology Division of Obstetrics and Feto‐Maternal Medicine Comprehensive Center for Pediatrics Medical University of Vienna Vienna Austria; ^7^ Exposome Austria Research Infrastructure and National EIRENE Node Vienna Austria

**Keywords:** infant, metabolomics, monocytes, premature, trained immunity

## Abstract

The survival of nonvertebrate species that lack adaptive immunity has prompted the discovery of memory mechanisms in innate immune cells, termed trained immunity. Because infants depend on innate immunity to fight off pathogens, leveraging these mechanisms in neonatal care may hold clinical significance. Yet, this is constrained by limited understanding of innate immune memory development during early‐life. By performing systems‐level characterization of β‐glucan‐trained monocytes from adults, term infants, and preterm infants, we report maturation‐dependent constraints in trained immunity. We corroborate these findings in a small cohort of premature infants with in utero pathogen exposure. Alongside limited stimulus‐induced reprogramming of innate immune responses in the clinical cohort, monocytes from premature infants exhibited metabolic alterations in glutaminolysis and tricarboxylic acid cycle metabolism, pathways central to trained immunity. They also displayed a treatment‐robust long noncoding RNA landscape and transcriptional alterations in enzymes relevant to epigenetic induction of innate immune memory. Finally, network‐based analyses implicated SMAD5 and ARNT as potential drivers of differences and provided evidence for altered transcriptional regulation of chromatin organization across early‐life development. Our results suggest that metabolic and transcriptomic immaturity constrains trained immunity in premature infants, highlighting a developmental mechanism that may affect host defense and guide future immunomodulatory strategies.

## Graphical abstract

In premature infants, innate immune memory may serve as an important compensatory mechanism for the underdeveloped adaptive immune system. However, monocytes derived from prematurely born individuals display abnormal metabolic and insufficient transcriptomic responses to β‐glucan‐induced training. Clinically, this is reflected by the limited effect of in utero pathogen exposure on subsequent cytokine responses to heterologous antigens.

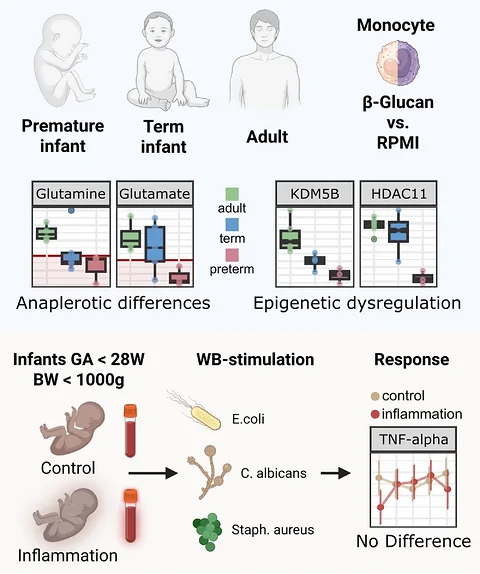

## 1 Introduction

Trained immunity, the long‐term propagation of functional changes in innate cells that have previously encountered an antigenic stimulus, challenges the categorization of immunity into adaptive and innate fractions [1]. The existence of innate immune memory in plants and species that lack adaptive immune cells has long been known [2]. Considering that adaptive immunity independently developed twice throughout vertebrate evolution (variable lymphocyte receptors and immunoglobulin‐dependent memory), the survival of nonvertebrate species devoid of adaptive immunity suggests that innate immune memory contributes to host–pathogen interactions [2].

Among the first cells to encounter antigens within the periphery, macrophages pose an attractive target for memory‐related effects. Exposure to pathogen‐associated molecular patterns, such as β‐glucan [3], bacteria like Bacillus Calmette‐Guérin (BCG) [4], or endogenous molecules like oxidized low‐density lipoprotein [5], alters monocyte reactivity upon restimulation. In contrast to adaptive immune memory directed toward a defined epitope, changes in cellular functions govern responses to novel antigens in trained immunity. Innate immune memory relies on initiation of distinct epigenetic and metabolic programs [6]. Regulation of long noncoding RNAs (lncRNAs), histone acetyltransferases (HATs), histone demethylases, and nicotinamide‐adenine‐dinucleotide‐dependent deacetylases alters chromatin accessibility through epigenetic imprinting [6, 7, 8]. Associated changes in transcriptional activity persist long beyond the initial stimulus. Imprinting efficacy is highly related to cellular metabolism. Metabolites like acetyl–CoA, α‐ketoglutarate, or succinate shape the activity of epigenetic regulators like HATs, ten‐eleven translocation (TET) enzymes, and Jumonji C‐domain‐containing (JMJD) proteins [9, 10, 11, 12, 13]. These delicate interactions, capable of introducing long‐lasting changes in cytokine responses of innate cells, suggest that perturbations in any of these pathways influence a host's ability to initiate trained immunity. Such functional deficits might hold clinical relevance during pathogen encounters.

Chorioamnionitis and intrauterine inflammation are major contributors to preterm birth and are associated with early‐life morbidity and long‐term sequelae [14]. Beyond triggering premature delivery, antenatal inflammation may shape fetal immune development during a period in which innate cells are still undergoing maturation. Inflammatory insults are thought to predispose infants to inflammation‐linked complications like bronchopulmonary dysplasia or necrotizing enterocolitis [15]. However, whether perinatal inflammation leaves an imprint on innate immune responsiveness to heterologous restimulation remains enigmatic.

Alterations in glycolytic activity and chromatin accessibility in neonatal monocytes/macrophages suggest that perturbations relevant to trained immunity may exist at birth [16, 17, 18]. Ehlers et al. [19] showed that neonatal monocytes exhibit enhanced oxidative phosphorylation to support postnatal myeloid differentiation, whereas increased glycolytic activity during early childhood was associated with greater inflammatory responsiveness. Congruently, impaired glycolytic and mammalian target of rapamycin (mTOR)‐dependent metabolic reprogramming may occur in preterm monocytes after inflammatory stimulation [17, 19, 20], suggesting that immunometabolic constraints may limit pathogen responses and innate immune memory. These alterations might hold ontogenetic significance throughout the third trimester and contribute to physiological immaturity of innate cells in premature infants. However, durable epigenetic imprinting has been observed in monocytes of term‐neonates after BCG vaccination [21], suggesting that neonatal cells retain responsiveness to immune training. Whether this functionally resembles the response of adults is unclear, as data suggest that BCG‐trained newborn monocytes develop a tolerogenic response to lipopolysaccharide (LPS) restimulation [22]. Thus, how monocytes from premature infants respond to innate immune training remains unknown.

Employing the well‐established experimental setup of β‐glucan training of primary monocytes [5, 11, 13, 23], we performed a comprehensive analysis of trained immunity throughout early‐life development. Our data suggest that stimulus‐induced reprogramming of monocytes by β‐glucan is reduced throughout early‐life development, especially in premature infants. This is supported by maturation‐associated alterations in baseline metabolic function and differential metabolic responses of term and preterm monocytes to β‐glucan. Additionally, transcriptomic alterations in genes involved in the metabolic and epigenetic responsiveness to immune training, as well as distinct maturation‐associated patterns, were identified.

## 2 Results

### 2.1 Monocytes Isolated From Cord Blood of Preterm Infants Display Altered Reprogramming of Cytokine Responses Upon Innate Immune Training

To assess whether innate cells of premature infants exhibit perturbations capable of constraining innate immune memory, we used the established model of β‐glucan training of primary monocytes and analyzed immune responses throughout early‐life maturation (Figure 1A).

**FIGURE 1:**
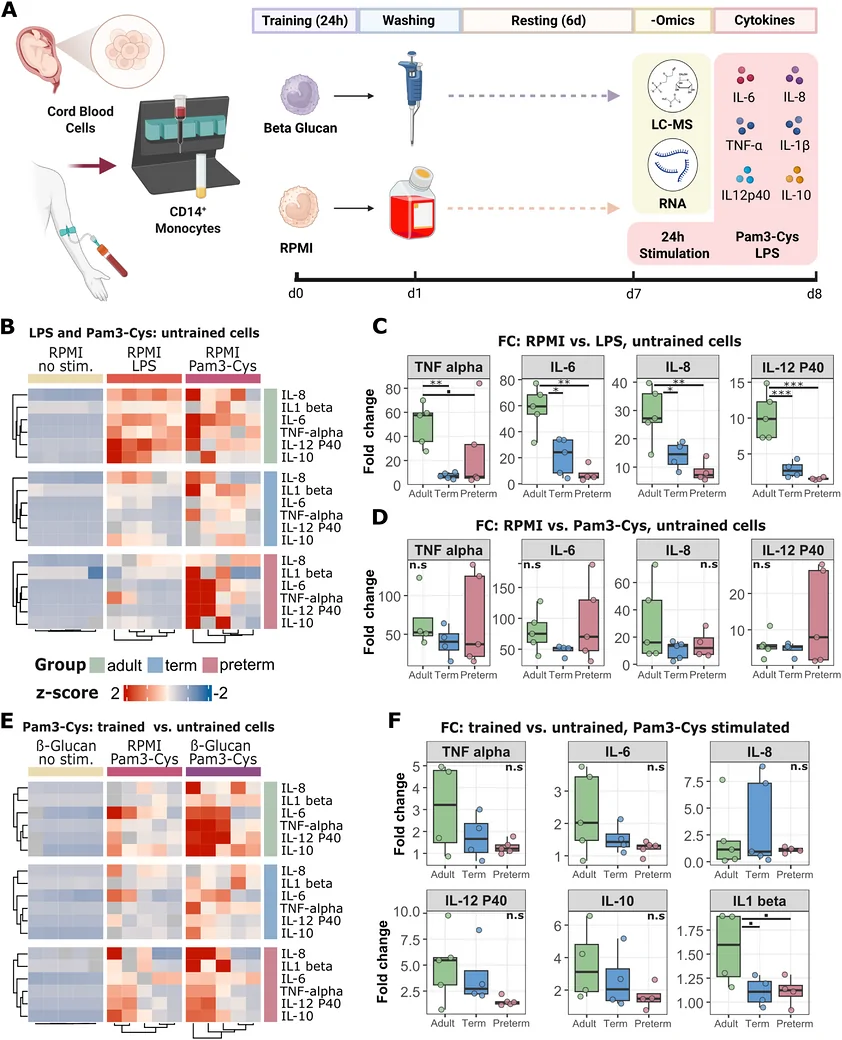
Monocytes derived from premature infants display minimally altered cytokine responses after training with β‐glucan and restimulation with Pam3‐Cys. Monocytes derived from adults, term infants, and premature infants were stimulated for 24 h with 5 µg/mL β‐glucan, allowed to rest for 6 days in RPMI medium, and subjected to functional cytokine assays, targeted LC–HRMS, or paired‐end RNA‐seq analyses (A). *Z*‐scaled cytokine concentrations measured from the supernatant of untrained control monocytes and untrained monocytes stimulated with 10 ng/mL LPS or 10 µg/mL Pam3‐Cys for 24 h after 6 days of resting (B). Fold changes in cytokine secretion between untrained controls and LPS (C) or Pam3‐Cys‐stimulated (D) untrained monocytes for adults (green, *n* = 4–5), term infants (blue, *n* = 4–5), and preterm infants (red, *n* = 4–5). *Z*‐scaled cytokine values observed in the supernatant of trained monocytes after 6 days of resting, restimulation of untrained monocytes with 10 µg/mL Pam3‐Cys for 24 h, or restimulation of trained monocytes with 10 µg/mL Pam3‐Cys for 24 h (E). Comparison of fold changes in cytokine secretion of trained and untrained monocytes restimulated with 10 µg/mL Pam3‐Cys for 24 h after 6 days of resting between adults (green, *n* = 4–5), term infants (blue, *n* = 4–5), and premature infants (red, *n* = 4–5) (F). •*p* < 0.1; *p < 0.05; **p < 0.01; ***p < 0.001.

Increased secretion of proinflammatory cytokines such as tumor necrosis factor alpha (TNF‐α) or interleukin‐6 (IL‐6) after heterologous restimulation with neoantigens is a hallmark of innate immune training [23, 24]. Although β‐glucan‐induced trained immunity has been thoroughly investigated in adult cells, work on BCG‐trained newborn monocytes indicates that neonatal innate immune reprogramming follows a different trajectory [22]. Therefore, we tested whether term and preterm monocytes display adult‐like cytokine responses after β‐glucan training and heterologous restimulation. Studies in adults have investigated immune training via Toll‐like receptor (TLR)‐4 and TLR‐1/2 stimulation [5]. However, monocyte TLR function varies during early‐life development [25, 26]. Thus, we compared the potency of the triacylated lipopeptide Pam3‐Cys and LPS exposure at baseline across groups. Comparison of cytokine expression values revealed reduced responsiveness of term and preterm monocytes to LPS‐induced activation (Figure 1B,C). In contrast, TLR1/2 agonist Pam3‐Cys displayed comparable proinflammatory efficacy across groups (Figure 1D). Consequently, Pam3‐Cys was used as the stimulatory agent in subsequent experiments. Comparison of *Z*‐scaled cytokine expression values of stimulated trained and untrained monocytes suggested reduced efficacy of innate immune memory in neonatal cells (Figure 1E). Although statistically constrained due to scarcity of preterm cord blood, maturation‐dependent trends in training efficacy were observed across the groups (Figure 1F). Absolute cytokine concentrations are reported in Figure S1.

The results underline differences in TLR signaling between adult and neonatal monocytes [25, 26] and suggest reduced ability of cultured cells to respond to β‐glucan‐induced immune training during early‐life.

### 2.2 Metabolic Imprinting by Transient In Vitro β‐Glucan Stimulation Varies During Early‐Life Maturation

Metabolism is a key regulator of trained immune responses in human monocytes/macrophages [11, 12, 13]. Alterations in glycolysis, tricarboxylic acid cycle (TCA), cholesterol metabolism, and β‐oxidation influence the function of epigenetic regulators of trained immunity via interactions with intermediary metabolites [6]. Differences in the activity of mTOR‐dependent energy pathways have been reported in monocytes derived from the umbilical cord blood (UCB) of premature infants [17]. Moreover, metabolism of neonatal monocytes is likely poised for postnatal myeloid differentiation, potentially limiting metabolic responses to exogenous stimuli [19]. We used our in vitro model to explore whether metabolic imprinting elicited by transient β‐glucan exposure differs between adult, term, and preterm monocytes after the resting/differentiation phase.

A total of 52 metabolites were quantified by liquid chromatography–high‐resolution mass spectrometry (LC–HRMS) from trained and untrained monocytes (Table S1). Metabolites absent in Figure 2 are included in Figure S2. Multilevel principal component analysis (PCA) revealed differences in metabolic responses to β‐glucan between monocytes derived from adults, term, and preterm infants (Figure 2A). Uniformity of treatment effects was dependent on biological age, with monocytes from preterm infants displaying the most disparate profile upon β‐glucan treatment. These effects were absent in conventional PCA (Figure S3A), indicating that interindividual variability represented the dominant source of variance in the unadjusted data. While reduced glycolytic activity was reported for polarized monocyte‐colony‐stimulating factor (M‐CSF)‐treated neonatal UCB‐derived macrophages [16], our data on unpolarized cells cultured without M‐CSF do not exhibit such a baseline reduction (Figure S3B, red). Rather, metabolite concentrations in the absence of β‐glucan argue for differential baseline activity of mitochondrial respiration (Figure S3B, blue), arginine metabolism (Figure S3B, yellow), and glutaminolysis (Figure S3B, green) between adult and neonatal cells. Differences in experimental setup, particularly M‐CSF‐driven cellular polarization, may account for these discrepancies and limit direct comparability. Glutaminolysis is central to trained immunity [11]. Inverse changes in glutamine (*p* < 0.1) and glutamate (*p* < 0.05) abundance between adult and preterm monocytes in response to β‐glucan suggest perturbation of this pathway in preterm cells (Figures 2B and S3B, green). Since glutaminolysis feeds into the TCA through deamination, we determined α‐ketoglutarate, succinate, and fumarate abundance. These metabolites are regulators of dioxygenases with demethylase activity at promoters of proinflammatory genes and control the activity of enzymes mediating degradation of hypoxia‐inducible factor‐1α (HIF‐1α) [6, 10, 27]. We observed alterations in α‐ketoglutarate (*p* < 0.1) and fumarate (*p* < 0.1) responses to β‐glucan across early‐life development (Figure 2B, blue), with cells from term infants displaying an intermediate profile. Glutamine may ultimately be converted to malate, influencing cytosolic pyruvate concentrations and the compartmentalization and metabolism of aspartate [27, 28]. We observed inverse β‐glucan‐dependent changes in malate (*p* < 0.01) and aspartate concentrations (*p* < 0.1) between treated monocytes from premature infants and adults (Figure 2B, blue/gray). As a central constituent of the aspartate–argininosuccinate cycle, aspartate contributes to fumarate and arginine accumulation [27, 28]. While fumarate concentrations were altered between groups, arginine was comparatively unaffected by immune training (Figure 2B, yellow).

**FIGURE 2:**
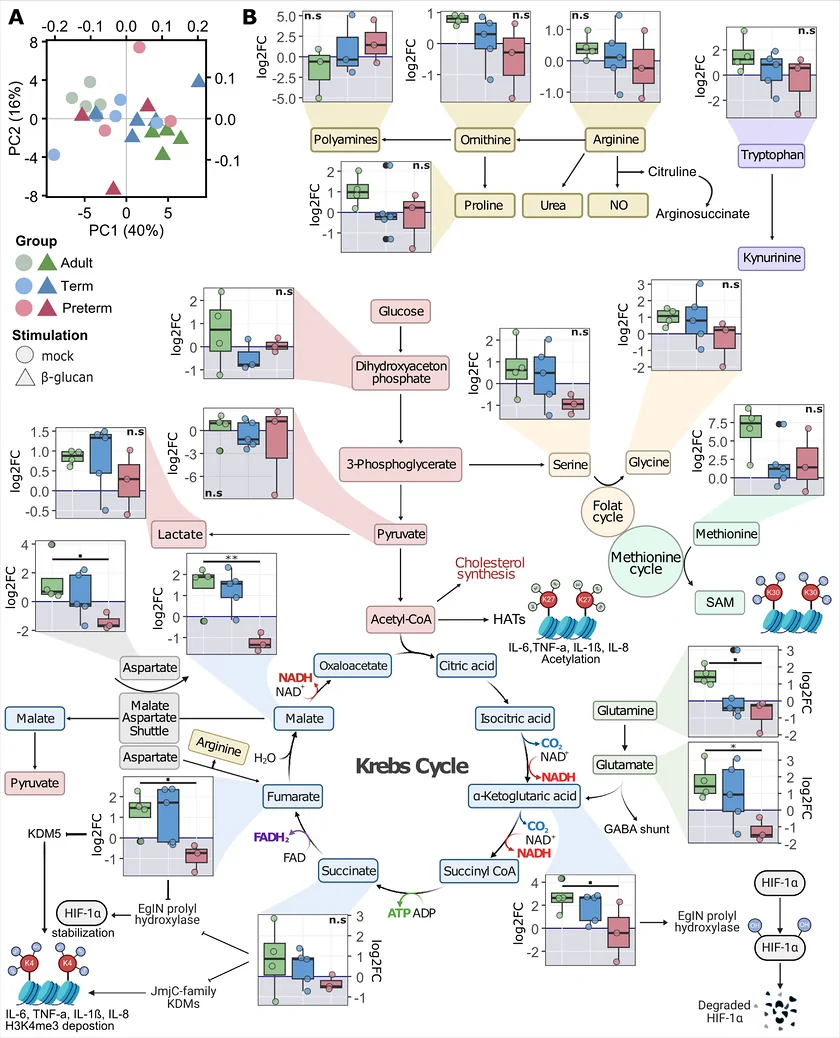
Metabolic responses to β‐glucan training in human monocytes differ across maturation. Multilevel principal component analysis was performed to compare the global metabolomic profile of β‐glucan‐treated (triangles) and mock (circles) monocytes derived from adults (green, *n* = 4), term infants (blue, *n* = 5), and premature infants (red, *n* = 3) (A). Log2‐fold changes of metabolite concentrations after training with 5 µg/mL β‐glucan for 24 h and resting for 6 days are shown for adults (green, *n* = 4), term‐born infants (blue, *n* = 5), and preterm infants (red, *n* = 3) (B). Results have been integrated into a metabolic framework based on previously published data [9, 10, 11]. For reasons of simplicity, we chose not to depict subcellular spaces (cytosol and mitochondrial matrix) in our graphical representation. •*p* < 0.1; *p < 0.05; **p < 0.01; ***p < 0.001.

The data suggest maturation‐associated alterations in the activity of anaplerosis, aspartate–argininosuccinate metabolism, and TCA in human monocytes cultured in vitro, especially in response to β‐glucan exposure. The divergent patterns between adult and preterm cells could point to ontogeny‐associated differences in β‐glucan responsiveness. Conclusions regarding broader in vivo metabolism will require validation in complex models.

### 2.3 Neonatal and Adult Monocytes Differ in Their Transcriptomic Response to β‐Glucan

In adult monocytes, mRNA levels of key‐metabolic enzymes [29] differ between trained and untrained monocytes after β‐glucan treatment [11]. Therefore, we performed transcriptomic analyses of β‐glucan effects throughout early‐life development to investigate alterations in gene expression relevant to immune training.

Throughout all six possible comparisons, RNA‐seq revealed a total of 963 differentially expressed genes (DEGs; Figure 3A and Supporting Information S1). Only adult monocytes displayed consistent transcriptomic responses to β‐glucan with 75 genes upregulation and 53 genes downregulated. Although ontogeny‐dependent transcriptomic differences between adult and neonatal cells were observable at baseline (138 upregulated, 113 downregulated), they markedly increased upon β‐glucan treatment (339 upregulated, 221 downregulated), suggesting inverse regulation. PCA of all samples revealed age rather than treatment as the dominant source of variance (Figure 3B). However, inverse responses of adult and preterm cells to β‐glucan observable in the DEG analysis propagated into better ontogeny‐related separation of transcriptomic profiles in treated samples (Figure 3C). In line with global PCA, hierarchically clustered DEGs largely followed group‐dependent trajectories with no clustering related to β‐glucan treatment (Figure 3D). Due to the absence of treatment‐related patterns in neonatal cells, we investigated whether the 128 upregulation or downregulated genes after β‐glucan treatment in adult cells confer biological functionality. Overrepresentation analysis (ORA) revealed alterations in cellular respiration, transmembrane transport, and calcium signaling (Figure 3E). During manual curation of genes included in the biological theme comparison, changes in the expression of transcripts related to amino acid transport (SLC7A8), respiratory chain activity (MT‐ND2, Cytochrome B, MT‐CO3) and mitochondrial calcium uptake (SLC25A23) (Figure 3F) were noted. Gene‐set variation analysis (GSVA; Figure S4) revealed significant (*p*. adj. < 0.05) downregulation of pathways involved in reactive oxygen species and fatty acid metabolism, as well as a tendency (*p*. adj. < 0.1) for downregulation of oxidative phosphorylation and glycolysis in adult cells upon treatment with β‐glucan. No statistically significant β‐glucan effect on neonatal cells was observed.

**FIGURE 3:**
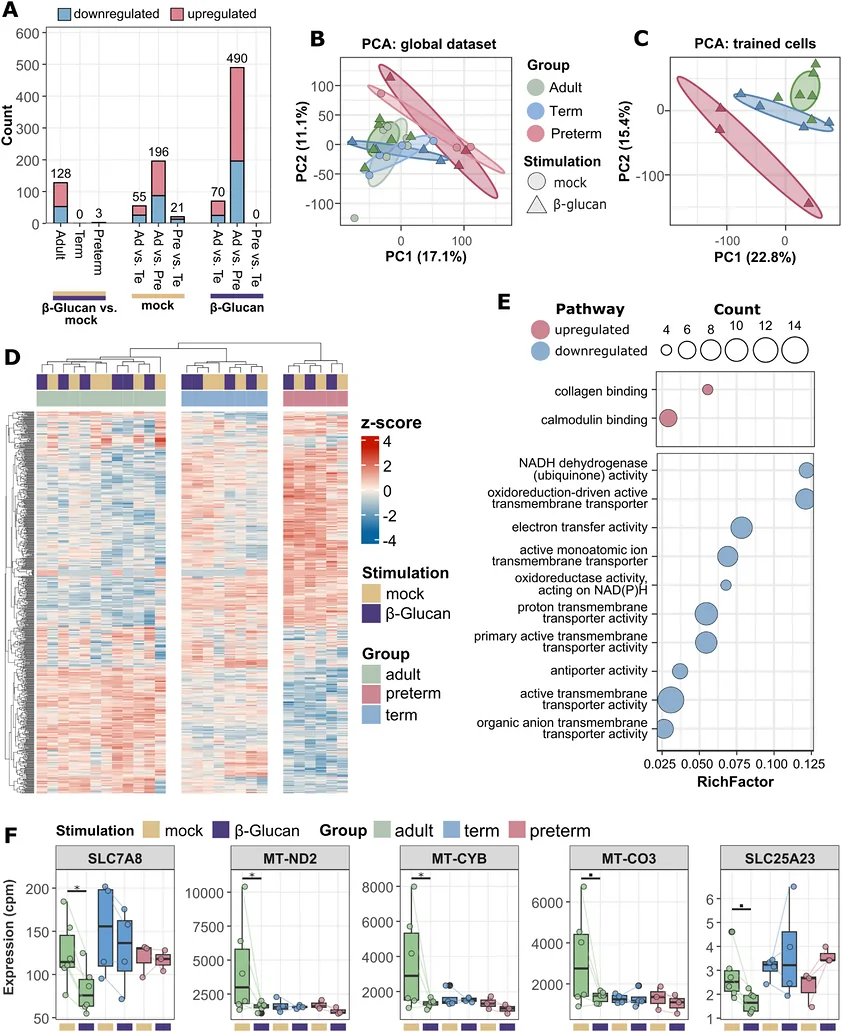
Maturation‐dependent differences in transcriptional responses of monocytes to transient β‐glucan stimulation. Number of differentially expressed genes with an adj. *p*‐value < 0.05 and a log2FC > 0.58 or <−0.58 displayed as a bar chart for all pairwise comparisons (A). Principal component analysis (PCA) depicting transcriptional similarities and differences between untrained and β‐glucan‐trained monocytes derived from adults (green, *n* = 6), term infants (blue, *n* = 4), and preterm infants (red, *n* = 3) for the full dataset (B) and β‐glucan‐trained cells only (C). Hierarchical clustering of differentially expressed genes deriving from all pairwise comparisons in the dataset (D). Overrepresentation analysis of DEGs from the comparison between trained and untrained adult monocytes (E). Comparison of the expression of biologically interesting genes (adj. *p*‐value < 0.01) that contribute to observed alterations in differentially active pathways in adults, across treatments (untreated = beige, β‐glucan = violet) and groups (adults = green, term infants = blue, preterm infants = red) in counts per million (F). •*p* < 0.1; *p < 0.05; **p < 0.01; ***p < 0.001.

Although our data suggest limited β‐glucan‐induced transcriptomic alterations after 6 days of resting, absence of treatment‐related effects in neonatal monocytes in the presence of altered transcription of metabolically relevant genes in adults is evident. The more than twofold increase in DEGs upon β‐glucan treatment of adult and preterm monocytes suggests inverse transcriptional activation, matching observed differences in metabolic activity.

### 2.4 Neonatal Monocytes Display Transcriptomic Perturbations at the Intersect Between Metabolome and Epigenome

To dissect differences in transcriptional responses between adult, term, and preterm monocytes at baseline and depict inverse regulation upon β‐glucan treatment, we retrieved DEGs with a log2‐fold change (log_2_FC) > 0.58 or <−0.58 and depicted them in a Venn diagram (Figure 4A). Genes of relevant intersects (Figure 4A) were annotated according to their relevance to monocyte biology and trained immunity. Gene‐set enrichment analysis (GSEA) was performed to investigate β‐glucan‐induced effects on neonatal cells.

**FIGURE 4:**
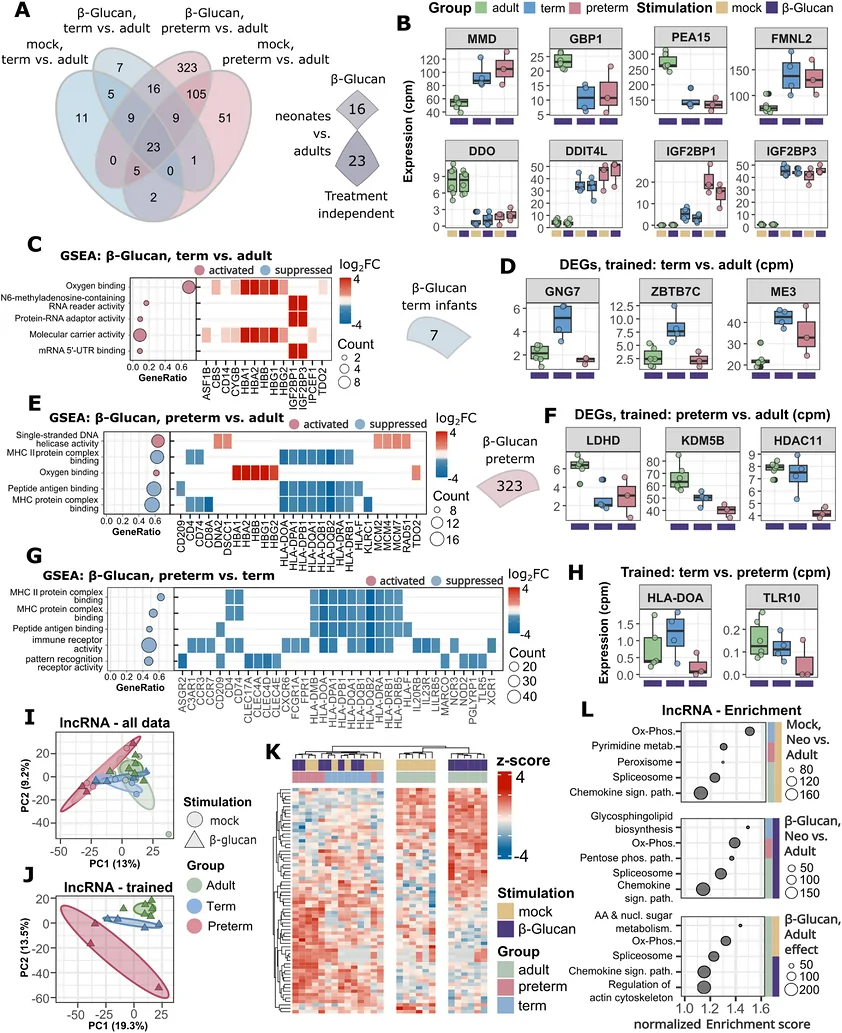
Transcriptomic perturbations at the intersect between metabolism and epigenetic regulation underlie differences in β‐glucan responses across human development. Intersects of differentially expressed genes with an adj. *p*‐value < 0.05 and a log2FC > 0.58 or <−0.58 between neonatal and adult cells shown in a Venn diagram (A). Expression of differentially expressed genes between neonatal (blue, red, *n* = 7) and adult (green, *n* = 6) cells occurring after treatment with β‐glucan (upper panel, B) or independent of treatment (lower panel, B). GSEA comparing the differential activity of pathways with an accompanying heatplot depicting genes with the highest log2FC for comparisons between β‐glucan‐treated term (*n* = 4) and adult (*n* = 6) (C), preterm (*n* = 3) and adult (*n* = 6) (E), and preterm (*n* = 3) and term (*n* = 4) monocytes (G and H). Expression of manually curated DEGs for each comparison depicted in counts per million (D and F). PCA comparing global long‐noncoding RNA (lncRNA) profiles of all samples (I) and only β‐glucan‐treated samples (J) between adult (green, *n* = 6), term (blue, *n* = 4), and preterm (red, *n* = 3) monocytes. Hierarchical clustering of differentially expressed lncRNAs across all comparisons (K). Network‐based analyses of the biological influence of differentially expressed lncRNAs between untreated neonatal and adult monocytes (upper panel), β‐glucan‐trained neonatal and adult monocytes (middle panel), and the effect of β‐Glucan on adult monocytes (lower panel) (L).

When focusing on conserved differences between adult and neonatal cells, 23 genes expressed at different abundances irrespective of treatment and 16 genes upregulation or downregulated only upon immune training were identified. The treatment‐independent fraction of DEGs included transcripts related to aspartate metabolism, mTOR‐function, and N^6^‐methyladenosine (m6A)‐mRNA stability (Figure 4B). When comparing β‐glucan‐treated term and adult monocytes, GSEA revealed upregulation of oxygen binding (Figure 4C). Intersect analysis indicated altered expression of malic enzyme, a mediator of the decarboxylation of malate to pyruvate (Figure 4D). GSEA identified differences in the pathway enrichment of DNA helicase activity and major histocompatibility complex (MHC)‐associated functions between treated preterm and adult monocytes (Figure 4E). Manual curation of DEGs revealed changes in metabolic and epigenetic regulators of trained immunity. Glycolytic enzyme LDH, amino acid transporters SLC7A6, SLC6A7, and SLC7A11, and histone demethylases JMJD3 and KDM5B differed in abundance (Figure 4F and Supporting Information S1). The latter constitutes an important epigenetic regulator of immune training [6]. Finally, we investigated differences in monocyte training between preterm and term infants. Again, downregulation of MHC‐related functions was observed in monocytes from premature infants (Figure 4G). This aligns well with HLA‐DR expression on monocytes following an inverse correlation with gestational age [30]. Chemokine and IL receptors, as well as pattern recognition receptors, were also downregulated in preterm cells (Figure 4G,H). However, differences between treated and untreated neonatal cells were not statistically significant (Figure 4H).

GSEA and manual annotation of DEGs revealed differences in expression of transcripts important for the metabolic and epigenetic regulation of trained immunity. This likely contributes to differences in β‐glucan responses between neonatal and adult monocytes. However, exploratory pathway and candidate‐gene analyses are hypothesis‐generating and require further experimental validation.

### 2.5 Analysis of lncRNA Expression in Neonatal and Adult Monocytes and Their Relationship to Immune Training

Recent evidence suggests that immune gene‐priming lncRNAs are involved in epigenetic reprogramming of β‐glucan‐trained monocytes [6]. Modulated by nuclear factor of activated T cells (NFAT) signaling, these lncRNAs facilitate persistent epigenetic changes [7] by harnessing the 3D structure of chromatin to aid deposition of H3K4me at relevant promoter regions.

By filtering our sequencing data, 2283 lncRNA transcripts were identified. PCA of lncRNA expression across all samples suggested age as the dominant source of variance (Figure 4I). After training, a nuanced ontogeny‐related separation of adult, term, and preterm profiles emerged (Figure 4J). Hierarchical clustering was performed on all differentially expressed lncRNAs to assess whether β‐glucan‐induced effects could be inferred from the data (Figure 4K). Training‐related lncRNA responses were identified, albeit largely confined to adult monocytes. Again, global lncRNA expression followed an age‐dependent trajectory, with term and preterm cells harboring distinct profiles. lncPath [31] was employed to identify changes in pathway activities regulated by interactions between lncRNAs and target genes (Figure 4L). Alterations in oxidative phosphorylation, RNA‐splicing, and chemokine signaling were identified between neonatal and adult cells in absence of β‐glucan treatment (Figure 4L, top). Training of monocytes promoted lncRNA‐mediated differences in pentose phosphate pathway (PPP) activity and glycosphingolipid synthesis between adult and neonatal cells (Figure 4L, middle). Training of adult cells resulted in alterations in amino acid and nucleotide sugar metabolism as well as oxidative phosphorylation (Figure 4L, bottom).

The results suggest the existence of maturation‐dependent lncRNA profiles in monocytes, potentially capable of influencing responses to β‐glucan. Moreover, they indicate a role for lncRNAs in the regulation of metabolic activities relevant to trained immunity, such as oxidative phosphorylation, PPP, and amino acid metabolism.

### 2.6 Maturation‐Dependent Transcriptional Alterations Predominate Over β‐Glucan‐Induced Differences

Conventional RNA‐seq analysis based on statistical testing of predefined comparisons [32, 33] neglects the sample‐specific correlation structure of genes. Weighted gene coexpression network analysis (WGCNA) has been applied to analyze the coexpression of genes by clustering similarly responding transcripts into modules [34]. Thus, we applied it to find correlation structures specific to monocyte maturation and trained immunity.

Because WGCNA requires sample sizes larger than 15 [34], we used all samples for network construction. We were able to identify 20 modules of coexpressed transcripts (Figure 5A). Most modules were significantly correlated with group‐specific features rather than β‐glucan treatment (Figure 5B). We then performed ORA on module‐specific genes to only retain modules with annotatable biological function. MEM14, the only module associated with β‐glucan‐induced immune training, showed no clear biological signature. We refrained from further analysis of this module and added identified genes to Supporting Information S1. We then plotted the PC1 of biologically meaningful modules, the eigengene vector, in relation to age (Figure 5C) and identified several modules associated with maturation. These modules harbored genes relevant to nucleus and chromatin structure, immune signaling, cholesterol metabolism, and cytokine production (Figure 5D). We then identified transcripts with the strongest Pearson correlations with their respective module eigengenes (Figure 5E). This revealed high expression of DTL and MCM10 in preterm monocytes, molecules that interact with the transcriptional machinery and regulate heterochromatin assembly [35, 36, 37, 38]. Neonatal cells also displayed decreased levels of ETS‐1, a regulator of NF‐κB‐signaling and cytokine expression [39, 40]. For cholesterol metabolism‐related networks, MAFF, a proposed inflammation‐dependent controller of cholesterol uptake [41], and ATF3, a regulator of cholesterol 25‐hydroxylase [42], showed high module membership. As for cytokine‐ and cell surface receptor‐related functions, we identified BLNK, a molecule involved in antifungal responses of macrophages [43], and heparanase, an endo‐β‐glucuronidase and inducer of cytokine responses [44], as hub genes. As an orthogonal approach, we also constructed a coexpression network using the hCoCena framework [45] (Figure S5). Three coexpression modules associated with maturation were identified. No clear β‐glucan‐associated hub was observed. In line with Ehlers et al. [19], module M1 (1584 genes), a module associated with prematurity, was enriched for oxidative phosphorylation and cell‐cycle‐related processes.

**FIGURE 5:**
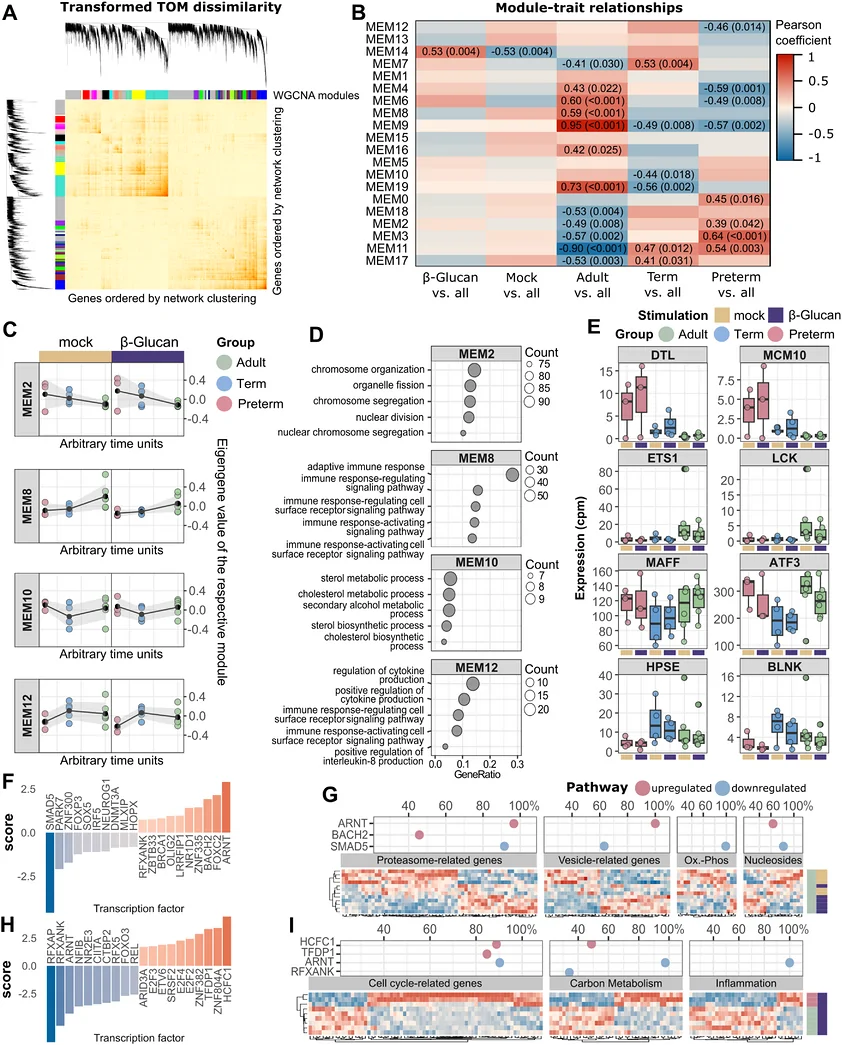
Network‐based analyses identify transcriptional modules related to monocyte maturation and reveal potential drivers of altered responses to immune training in neonatal monocytes. Dissimilarity matrix of the 10.000 most variable genes in our dataset across all samples with color intensity reflecting the degree of topological overlap between gene pairs (A). Association between identified modules and traits of interest, depicted as a heatmap with Pearson correlation coefficients indicated for significant (*p* < 0.05) interactions (B). Eigengene values of biologically relevant modules in relation to maturation, with arbitrary metric values reflecting the age of adult (green, *n* = 6), term infants (blue, *n* = 5), and premature infants (red, *n* = 3) (C). Dotplot depicting the overrepresentation analysis of genes included in modules of interest (D). CPM‐corrected expression of hub‐genes for each module in relation to group (adult = green, term infants = blue, premature infants = red) and treatment (mock = beige, β‐glucan = violet) (E). Barchart depicting results of transcription factor enrichment using DecoupleR with arbitrary scores representing the upregulation (red) and downregulation (blue) of regulatory molecules for treated adult (*n* = 6) cells (F) and the comparison between treated adult (*n* = 6) and preterm (*n* = 3) cells (H). Regulatory factors were reverse mapped to the database TF‐link and differentially expressed genes subjected to overrepresentation analysis. Results were plotted as a heatmap for the effect of β‐glucan treatment on adult cells (*n* = 6) (G) and differences between treated adult (*n* = 6) and preterm (*n* = 3) cells (I). The contribution of a transcription factor to a biological function was calculated and depicted as a dotplot using the percentage of genes regulated by the factor as a metric for pathway impact (G and I).

These results support the observation of nuanced β‐glucan‐induced and pronounced age‐dependent transcriptomic differences in monocytes. The existence of distinct maturation‐associated coexpression modules suggests differences in baseline responsiveness of neonatal monocytes to β‐glucan. Larger sample sizes would be required to test this hypothesis using consensus networks.

### 2.7 Network‐Based Analyses Identify Potential Transcriptional Drivers of β‐Glucan Effects in Monocytes Isolated From Adults and Neonates

To ascertain the underlying transcriptional regulation of observed differences, we used a network‐based approach introduced in the decoupleR framework [46] and, for validation, reverse‐mapped identified transcriptional regulators to their target genes using TF‐link [47].

ARNT, FOXC2, and BACH2 were positively associated with β‐glucan‐induced changes in adults, whereas activity of SMAD5, PARK7, and ZNF300 was reduced by immune training (Figure 5F). Reverse mapping revealed ARNT and SMAD5 to be involved in the regulation of proteasomal function, vesicle induction and nucleoside metabolism (Figure 5G). Downregulation of SMAD5 activity was related to oxidative phosphorylation, identifying the molecule as a possible regulator of metabolic responses to β‐glucan in adult monocytes (Figure 5G). In comparing β‐glucan responses between monocytes from premature infants and adults, our analysis revealed HCFC1, ZNF804A, and TFDP1 as positively associated and RFXAP, RFXANK, and ARNT as negatively associated with transcriptional differences observed in premature infants (Figure 5H). Activity of reverse‐mapped genes was related to cell cycle regulation (ARNT, HCFC1, TFDP1), metabolism (ARNT, HCFC1, RFXANK), and inflammation (ARNT) (Figure 5I). Considering the reciprocal relationship between the enhanced activity of ARNT in treated adult monocytes and its reduced activity in the control of genes differentially expressed between treated monocytes derived from premature infants and adults, ARNT might be considered a target molecule for further analysis of β‐glucan effects across maturation.

The analyses identify SMAD5 as a potential transcriptional regulator of metabolic effects of β‐glucan in adults and provide evidence for the involvement of ARNT in the development of maturational differences in trained immunity.

### 2.8 Exploratory Investigation of the Impact of In Utero Pathogen Exposure on Cytokine Responses to Restimulation in a Small Cohort of Premature Infants

Long‐lasting innate immune memory has been reported in BCG‐vaccinated neonates [21]. This has initiated a discussion on immune training in the context of intensive care for premature infants. We aimed to investigate the effect of in utero pathogen exposure on subsequent immune responses in premature infants ex vivo. The following analyses are not aimed at verifying the experimental findings in vivo, as direct clinical translatability is limited by the experimental model. They should be seen as a pilot study to determine whether inflammatory insults could serve as a training stimulus for subsequently altered immune reactivity.

We stratified infants recruited in our PreMiBraIn study [48] into those who likely experienced pathogen exposure in‐utero and noninflamed controls. Peripheral blood was sampled throughout the clinical course of admission and cytokine values after stimulation with heat‐killed bacteria (Figure 6A) were compared. Because diagnosing early‐onset sepsis in premature infants by blood culture is challenging due to limited sample volumes and peripartal antibiosis, children were classified into the “in utero exposure” cohort if either C‐reactive protein (CRP) exceeded 2.2 mg/dL or IL‐6 exceeded 1000 pg/mL within the first 3 days of life. Infants with both CRP and IL‐6 values consistently below 0.5 mg/dL and 150 pg/mL were assigned to the control group. An overview of the cohort is provided in Table 1 and differences in peak CRP and peak IL‐6 between groups are shown in Figure 6B. To allow for comparisons across timepoints we used linear mixed models [49] and corrected for gestational age and within‐sample variation, two well‐established sources of variance. Although multiple factors (e.g., antibiotic exposure, nutrition, interventions) may further influence immune development, we refrained from including additional covariates to avoid model overfitting. We then calculated the estimated marginal means for respective cytokines and stimulations. No significant differences in cytokine responses were observed for comparisons beyond Day 3 of life (Figure 6C). However, on Day 3 of life, significant differences in IL‐1β and TNF‐α responses in infants who experienced pathogen exposure in utero were observed. This indicates that our setup may have captured measurable variation in immune responsiveness.

**FIGURE 6:**
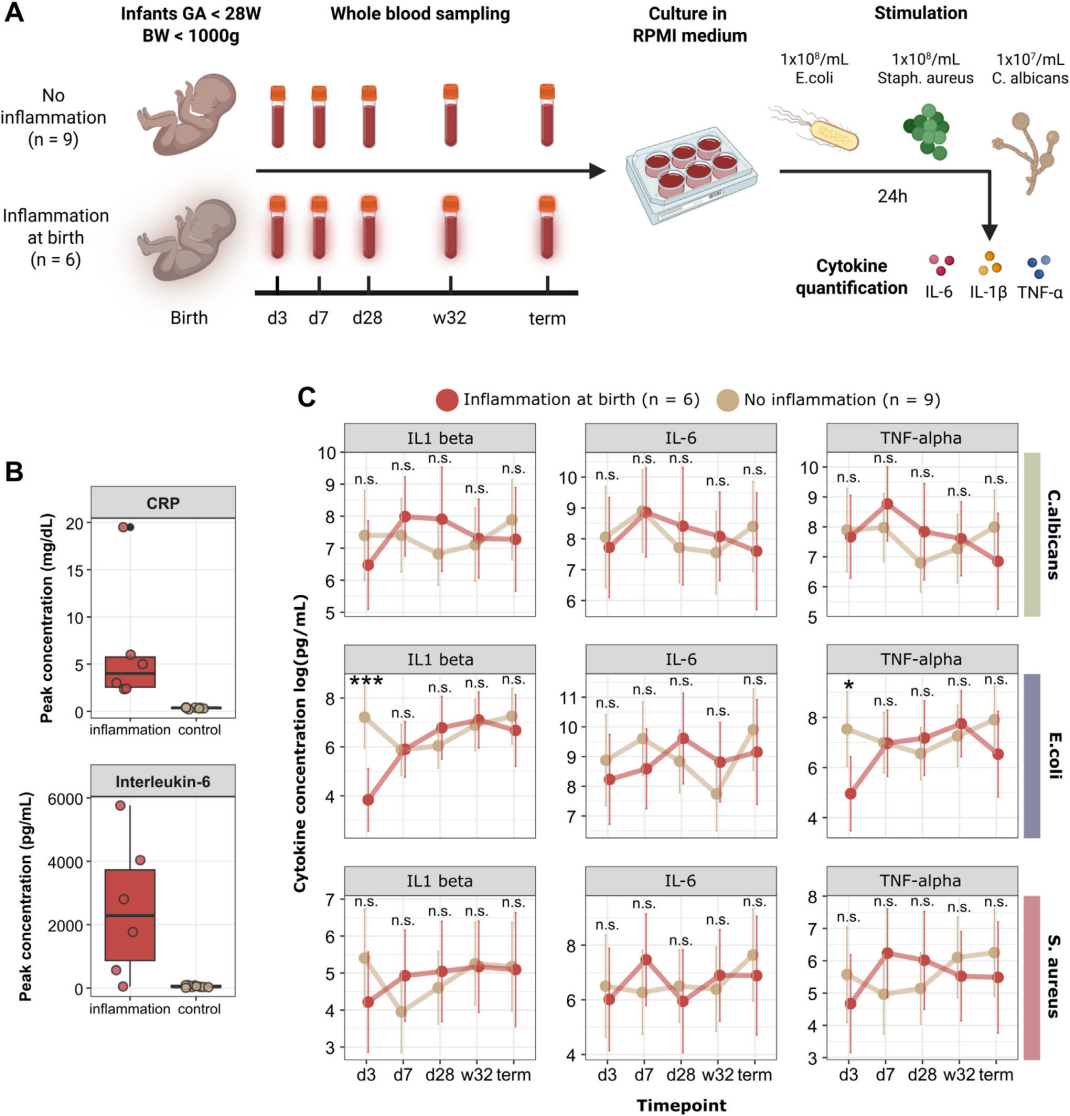
Analysis of the influence of in‐utero pathogen exposure on cytokine responses to heterologous restimulation with heat‐killed pathogens in premature infants. Premature infants were categorized into two groups based on whether they showed dysregulated CRP and IL‐6 values within the first 3 days of life. Blood was sampled longitudinally, and cytokine responses analyzed after restimulation for 24 h with heat‐killed E. coli (HKEC, 1 × 10^8^), Staph. aureus (HKSA, 1 × 10^8^), or Candida albicans (HKCA, 1 × 10^7^) (A). Highest CRP (top) and IL‐6 values (bottom) measured in routine clinical lab work within 3 days after birth for infants included in the in‐utero exposure (red, *n* = 6) and control (beige, *n* = 9) group (B). Cytokine concentrations (log10, pg/mL) in the supernatant of restimulated blood depicted as model‐derived marginal means with 95% confidence intervals over the course of maturation between in‐utero exposed (red, *n* = 6) and unexposed (beige, *n* = 9) infants (C). •*p* < 0.1; *p < 0.05; **p < 0.01; ***p < 0.001.

**TABLE 1:** Overview of baseline characteristics of the study cohort based on the presence or absence of in utero pathogen exposure. Continuous variables are given as mean ± standard deviation (SD). Distribution of categorical variables is displayed as percentages or counts.

	In utero exposure (*n* = 6)	Noninflamed controls (*n* = 9)
Sex (male/female)	4/2	2/7
Gestational age (weeks)	26 ± 1	26 ± 1
Birth weight (g)	756 ± 113	729 ± 161
Discharge weight (g)	2770 ± 505	2550 ± 385
APGAR score at min 10 (min–max)	4–9	9–9
Bronchopulmonary dysplasia (%)	33.3%	22.2%
Necrotizing enterocolitis (%)	0.00%	0.00%
Retinopathy of prematurity (%)	83.3%	44.4%
Max. CRP ≤ 3d after birth (mg/dL)	6.4 ± 6.6	0.3 ± 0.1
Max. IL‐6 ≤ 3d after birth (pg/mL)	2500 ± 2160	52.4 ± 31.0

Although not arguing for complete absence of effects on heterologous cytokine responses, the data suggest a limited effect size of in utero pathogen exposure/inflammation at the time of birth on subsequent cytokine responses in premature infants.

## 3 Discussion

Functional plasticity of immune responses is a prerequisite for successful host defense. The ability to alter immune responses upon secondary infection has independently been selected for twice during vertebrate evolution [2]. Previously thought to be intrinsic to adaptive immunity, it is now clear that altered responses to secondary antigenic stimuli also occur in innate cells. Considering the rapid transition between sterility in utero and nonsterility upon caesarean section, premature infants might benefit from targeting such innate mechanisms [50]. By performing a system‐wide analysis of in vitro β‐glucan‐treated monocytes isolated from prematurely born infants, term infants, and adults, we identified several perturbations in metabolic and transcriptional responses relevant to trained immunity.

Monocytes from premature infants display reduced H3K4me3 marks at promoter regions of immunologically relevant genes [18]. Our analysis of immune responses after training with β‐glucan, a process known to induce H3K4me3 deposition at relevant genes [6], revealed an inverse correlation between plasticity in cytokine expression and maturation (Figure 1). The T. versicolor‐derived β‐glucan particles used in this study induce trained immunity via Dectin‐1 binding, internalization, and activation of the AKT/mTOR/HIF‐α axis [20, 51]. Alterations in these processes might contribute to changes in immune plasticity observed in neonatal monocytes. Indeed, the function of mTOR, an inducer of HIF‐α activity [20], hence the metabolic induction of trained immunity [29], is altered in UCB‐derived macrophages [16].

LC–HRMS analyses revealed maturation‐dependent differences in the abundance of metabolites that modulate epigenetic changes in trained monocytes (Figure 2). In adult monocytes, glutamine fuels the TCA upon inhibition of pyruvate dehydrogenase by HIF‐α‐associated mechanisms [10, 27]. Thus, alterations in anaplerosis observed upon immune training in preterm monocytes may reflect differences in metabolic adaptation. Moreover, our data suggest differences in the abundance of α‐ketoglutarate, fumarate, and malate between adult and neonatal cells in response to training—metabolites that affect TET enzymes, JMJD‐domain histone demethylases, and other KDMs [6]. These molecules contribute to trained immune responses by controlling the accessibility of genes via H3K4me3 demethylation (e.g., KDM5) [52], 5‐methylcytosine oxidation (TET enzymes) [53], and H3K27 demethylation (e.g., JMJD‐3) [8] at promoter regions. Moreover, α‐ketoglutarate and fumarate may also influence the activity of HIF‐α by enhancing (α‐ketoglutarate) or inhibiting (fumarate) the activity of prolyl hydroxylases, enzymes that induce the degradation of HIF‐α [6]. Although mTOR and HIF‐α signaling introduce metabolic changes upon Dectin‐1 engagement by β‐glucan, long‐term propagation of immunometabolic alterations depends on the nexus between metabolome and epigenome.

While differential activity of the mTOR/HIF‐α axis contributes to metabolic and epigenetic constraints observed in our in vitro model, attributing alterations in immune plasticity solely to this mechanism constitutes an oversimplification. Our transcriptomic analyses (Figures 3, 4, 5) revealed a treatment‐independent upregulation of DDIT4L, a potential regulator of mTOR‐activity, innate immunity, and mitochondrial function [54]. Additionally, upregulation of IGF2BP1 and 3, regulators of m6A‐modified mRNA stability and expression [55], was present in our data. RNA methylation may influence the metabolic and immunological function of innate cells [56]. Moreover, a distinct lncRNA profile was detectable in neonatal monocytes (Figure 4). LncRNAs might coordinate transcription through regulation of H3K4me deposition at immunologically relevant promoters [6, 7]. Of note, lncRNA responses to β‐glucan appear constrained in our in vitro model in neonatal cells, despite lncRNA induction being based on mTOR‐independent, NFAT‐dependent Dectin‐1 signaling [7, 57]. Given that differentially expressed lncRNAs in our dataset modulate functions relevant to immunity and metabolism, this raises the question of whether mTOR‐independent pathways contribute to observed differences. However, it is unclear whether our model adequately captures NFAT‐dependent pathways, as their initiation has been proposed to require specific particulate agonists [58].

Nevertheless, the differences identified in our experimental model at baseline and after β‐glucan treatment across maturation merely constitute one part of the picture. Soluble factors that comature with cellular immunity also shape innate immune functionality [59] and may affect the efficacy of trained immune responses [60]. Thus, transferability of results from studies on trained immunity conducted in adults and term‐infants to earlier stages of human development is limited. Our data on cellular differences suggest that the functional distinction of trainability in neonatal monocytes, especially those derived from premature infants, is based on both mTOR‐dependent and mTOR‐independent functions. While Dectin‐1‐mediated mTOR‐induced HIF‐α activity and associated metabolic adaptations seem impaired in premature infants due to reduced mTOR expression [16], this does not explain treatment‐independent differences in metabolism and immunological function observed at baseline. Fittingly, GSVA and hCoCena analyses indicated maturation‐dependent differences in metabolic pathway activity and transcriptional network organization at baseline. Oxidative phosphorylation‐associated programs were more pronounced in preterm monocytes, arguing that these cells do not merely display a deficient adult‐like response, but rather occupy a transcriptional‐metabolic state that preferentially engages in energy‐generating, maturational programs. This might limit metabolic flexibility for β‐glucan‐induced training. Maturation‐dependent expression of mitochondrial regulators like DDIT4L [54], epigenetic regulators like lncRNAs [7], and m6A‐mRNA stabilizers [55, 56] may be further reflections of this. However, how changes translate into physiology remains unclear.

To identify constraining factors of in vitro trainability of monocytes from premature infants, studies should focus on (I) signaling‐dependent aspects of reduced β‐glucan‐induced functional reprogramming via mTOR/HIF‐α inhibitors and varying Dectin‐1 ligands, (II) an analysis of lncRNAs and their functions, (III) investigation of metabolic flexibility of neonatal monocytes in response to training, and (IV) epigenetic alterations before and after proven infection in clinical cohorts. Such an approach would further our understanding of innate immune trainability in premature infants.

## 4 Conclusion

Our work provides initial insights that the capacity of human monocytes to initiate β‐glucan‐induced trained immunity is shaped by early‐life maturation. Compared with adult monocytes, neonatal and preterm monocytes displayed reduced β‐glucan‐induced functional reprogramming of immune responses together with maturation‐dependent alterations in metabolic, transcriptomic, and lncRNA‐associated pathways central to immune training. This suggests that the requirements for immune training, including coordinated metabolic reprogramming and epigenetic regulation, may not be fully developed during prematurity. Our proof‐of‐concept ex vivo data indicate that in utero inflammation exerts limited effects on subsequent heterologous cytokine responses in extremely premature infants. This emphasizes the need for further studies investigating the clinical impact of epigenetic, metabolic, and functional differences.

### 4.1 Limitations

A limitation of the present study constitutes the sample size in the monocyte cohort of premature infants. Observations ought to be validated and should be interpreted in a hypothesis‐generating manner, rather than a definitive physiological model. The isolation of sufficient numbers of CD14^+^ monocytes from UCB requires large quantities of blood (>10 mL). Such quantities are difficult to obtain from premature infants due to delayed cord clamping. Because acute cytokine responses to β‐glucan during the initial 24‐h training phase were not assessed, the contribution of maturation‐dependent differences in primary sensing of the training stimulus to reduced responses cannot be investigated. While allowing for direct comparison of cellular responses across maturation [61], the experimental design does not account for soluble factors [59, 60], as cells were cultured in adult human AB‐serum. Higher baseline variance in neonates may have contributed to the few DEGs observed in the transcriptomic analysis. Studies with ^13^C‐labeled glucose and glutamine were not performed. No definitive mechanistic conclusions can be drawn from the metabolomics data. As for the clinical analysis, we are aware that the absence of evidence does not indicate evidence of absence. The analysis does not indicate complete absence of stimulus‐induced functional reprogramming of innate immune responses but suggests a limited effect size of in utero pathogen exposure. Due to the low postpartum pathogen detection rate in blood cultures, in utero pathogen exposure was approximated through CRP and IL‐6 cutoffs. These cutoffs potentially allow for erroneous inclusion of nonexposed infants into the exposure group.

## 5 Materials and Methods

Detailed materials and methods can be found in Supporting Information.

### 5.1 Study Population and Ethical Approval

For whole blood cytokine assays, samples were obtained from extremely low birth weight infants born before 28 weeks of gestation within the framework of the *PreMiBraIn* study [48, 62], which includes information about inclusion and exclusion criteria and prospective patient monitoring. For monocyte assays, UCB of seven premature infants born between 28 and 34 weeks of gestation (female *n* = 3) and six term‐born infants (>37 weeks of gestation, female *n* = 3) was collected into heparin tubes (Greiner Bio‐One, Kremsmünster, Austria) from the placenta after caesarean section. Exclusion criteria were presence of maternal or neonatal infection, congenital malformations, or known genetic disorders. Blood was also collected from nine healthy adults between 18 and 35 years of age (female *n* = 4). The study was approved by the ethics committee of the *Medical University of Vienna* (*EC‐No. 1348/2017 and 2148/2017*). Written informed consent was obtained from all participants or their guardians.

### 5.2 Isolation of Monocytes and Cell Culture

Peripheral blood mononuclear cells were isolated using Ficoll‐Paque density gradient centrifugation and resuspended in Dulbecco's PBS (GIBCO, Carlsbad, CA, USA). CD14^+^ cells were isolated using magnetic bead separation (anti‐CD14 MicroBeads; Miltenyi Biotec, Bergisch‐Gladbach, Germany). Monocytes were transferred into a flat‐bottom 96‐well plate (Greiner Bio‐One, Kremsmünster, Austria) at a density of 500,000 cells/mL and cultured in 200 µL RPMI‐1640 medium (GIBCO) supplemented with 10% human AB‐serum (SeraCare Life Sciences Inc., Milford, MA, USA) [61]. Cells were kept in the presence or absence of 5 µg/mL β‐glucan (Invivogen, San Diego, CA, USA) for 24 h at 37°C and 95% humidity. Unless indicated otherwise, samples were then centrifuged at 300×*g* for 10 min, the supernatant was discarded, and monocytes rested in RPMI‐medium until further experiments. Under these conditions, monocytes are known to differentiate into macrophages. However, because β‐glucan training is initially conducted on monocytes whereas most downstream assays are performed on monocyte‐derived macrophages, we collectively refer to cells as “monocytes” and “trained monocytes,” or directly state the treatment conditions of the cells within the text.

### 5.3 Measurement of Monocyte and Whole Blood Cytokine Responses

β‐glucan‐treated and untreated monocytes were stimulated for 24 h with 10 ng/mL ultrapure LPS (Invivogen), 10 µg/mL Pam3‐Cys (Invivogen), or allowed to rest in fresh RPMI. Samples were centrifuged and cytokines quantified from supernatant using the Human Custom ProcartaPlex 8‐plex panel (Invitrogen, Waltham, MA, USA), according to manufacturer's instructions. Whole blood was sampled from premature infants at Day of life 3, 7, 28 as well as at a corrected gestational age of 32 weeks and at term‐corrected age. Samples were stimulated for 24 h with heat‐killed Escherichia coli (HKEC; 1 × 10^8^; Invivogen), heat‐killed Staphylococcus aureus (HKSA; 1 × 10^8^; Invivogen), or heat‐killed Candida albicans (HKCA; 1 × 10^7^; Invivogen) and cytokines quantified from supernatant using a Human Custom ProcartaPlex panel (Invitrogen, Waltham, MA, USA), according to manufacturer's instructions. For subsequent analyses, patients were stratified according to presence (CRP > 2.2 mg dL^−1^ or IL‐6 > 1000 pg mL^−1^) or absence (CRP < 0.5 mg dL^−1^ and IL‐6 < 150 pg mL^−1^) of inflammation within 3 days of birth.

### 5.4 Metabolomic Profiling of Monocytes by LC–HRMS

For the analysis a Vanquish ultra‐high‐pressure liquid chromatography system coupled to a QExactive HF quadrupole‐Orbitrap mass spectrometer via an electrospray ionization interface was used. A reverse phase (RP) column (Waters Acquity HSS T3; 1.8 µm, 100 × 2.1 mm) and a hydrophilic interaction liquid chromatography column (HILIC, SeQuant ZIC‐pHILIC; 5 µm, 150 × 2.1 mm) were employed. Measurements were conducted in full‐scan mode employing polarity switching. A pooled quality control was injected throughout the sequence and a reference standard mixture used for metabolite identification. The software Skyline (v. 20.2) [63] was used to process the resulting. raw files and to perform a suspect screening for the metabolites contained in the reference standard mixture.

### 5.5 RNA‐Extraction, Paired‐End Sequencing, and Gene Alignment

RNA was extracted from 1 × 10^6^ monocytes cultured under the conditions described above using the RNeasy Mini Kit (Qiagen, Hilden, Germany). PolyA‐enriched libraries were prepared at the Core Facility Genomics, Medical University of Vienna. Sequencing was performed on a NextSeq500 instrument (Illumina, San Diego, CA, USA) in 2×75 bp paired‐end mode, generating approximately 30 × 10^6^ read pairs per sample. After quality control [64, 65] and trimming [66], reads were aligned to the human reference genome (GRCh38, GENCODE v.45 [67]) using STAR v. 2.7.10a [68] with two‐pass mapping. Count matrices were generated using Rsamtools [69] and GenomicAlignments [70].

### 5.6 Differential Gene Expression and Enrichment Analyses

DEGs were identified using the limma framework [32]. Samples were normalized using trimmed mean of M‐values (TMM) [71] and the duplicate correlation function used to account for sample pairing. Genes with an adjusted *p*‐value (adj. *p*‐value) threshold below 0.05 and log2FC higher than 0.58 or lower than −0.58 were considered differentially expressed. DEGs with an adj. *p*‐value below 0.1 and a log2FC higher than 0.58 or lower than −0.58 were selected for ORA using the enrichGO function with default settings [72]. GSEA was performed using the gseGO function with molecular function selected as reference ontology [72, 73]. GSVA [74] was performed on variance stabilized genes using human hallmark gene sets from the Molecular Signatures Database (MSigDB) [75], and significance assessed via limma [32].

### 5.7 Network‐Based Analyses

Differentially expressed lncRNAs were identified by filtering the dataset for ENSEMBL‐annotated lncRNAs and pathway influence inferred using the lncPath framework [31]. The five biologically most relevant pathways with a false discovery rate < 0.05 were selected and depicted. WGCNA [34] was performed on the 10,000 most variable genes by constructing a signed network, identifying significant module‐trait relationships, inferring biological function of modules via ORA [72], extracting eigengene values from relevant modules and identifying respective module hub‐genes. As an orthogonal approach an undirected integrated network was calculated using hCoCena according to recommendations [76] and functional enrichment analyses performed using human hallmark gene sets from MSigDB [75]. Finally, transcriptional regulators were identified by mapping genes to the transcription factor‐target gene network CollecTRI using the decoupleR framework [46]. The 10 most differentially positively and negatively enriched transcription factors were visualized and reverse‐mapped to their gene targets using the TF‐link database [47] prior to ORA to infer biological functionality.

### 5.8 Statistical Analysis

For the longitudinal comparison of cytokine responses between preterm infants who presented with in utero pathogen exposure and noninflamed controls, linear mixed models with the formula log(cytokine) ∼ group × timepoint + gestational age + (1 | patient) were created. Estimated marginal means and 95% confidence intervals were calculated from the models for each respective timepoint, pairwise comparisons were performed and *p*‐values were Bonferroni corrected. For the analysis of cytokine and metabolic responses to immune training or LPS/ Pam3‐Cys stimulation across ontogeny, linear models were created with adult responses set as reference. *p* Values were extracted from the model coefficients and Bonferroni corrected. Supplementary metabolomics data were analyzed using a two‐way repeated measures ANOVA followed by pairwise *t*‐tests with Benjamini–Hochberg correction.

## Author Contributions

Conceptualization: AB, BW, DB, and LW. Methodology: ED, DS, and MP. Clinical sample acquisition: ED, DS, HK, AF, and MA. Investigation: ME, MP, and LW. Visualization: ME and LW. Supervision: LW. Writing – original draft: ME and LW. Writing – review and editing: DB, AB, BW, and LW. All authors have read and approved the final manuscript.

## Funding

The study was funded by in‐house funding of the Medical University of Vienna and the University of Vienna and supported by the Exposome Austria Research Infrastructure.

## Conflicts of Interest

The authors declare no conflicts of interest.

## Ethics Statement

The study was approved by the ethics committee of the *Medical University of Vienna* (*EC‐No. 1348/2017 & 2148/2017*). Written informed consent was obtained from all participants or their guardians.

## Supporting information




**Supporting File 1**: mco270939‐sup‐0001‐SuppMat.xlsx

**Supporting File 2**: mco270939‐sup‐0002‐SuppMat.docx

## Data Availability

Raw paired end sequencing data are available from the European Nucleotide Archive (ENA) under the accession number *PRJEB115678*. Metabolomics data are available from *Phaidra* under the link https://phaidra.univie.ac.at/detail/o:2070841. The code for respective analyses has been uploaded on *GitHub* and can be accessed via the link https://github.com/wisgrill‐lab/Maturation‐Dependent‐Constraints‐in‐the‐Ability‐of‐Human‐Monocytes‐to‐Initiate‐Trained‐Immunity.
