# Supplementary material for: Maturation‐Dependent Constraints in the Ability of Human Monocytes to Initiate Trained Immunity
Source: MedComm (2020). 2026 Oct 4;7(10):e70939. doi: 10.1002/mco2.70939 (PMC13634178; doi:10.1002/mco2.70939)
Supplement: Supplementary file 1 [file MCO2-7-e70939-s002.docx]

Supporting Information for

Maturation-Dependent Constraints in the Ability of Human Monocytes to Initiate Trained Immunity

Michael Eigenschink^1,2^, Elsa Davogg^1,2^, Manuel Pristner^3^, David Seki^4,5^, Marcus Arzmüller^1,2^, Herbert Kiss^2,6^, Alex Farr^2,6^, Angelika Berger^1,2^, David Berry^4,5^, Benedikt Warth^3,7^, Lukas Wisgrill^1,2,7^*

^1^Department of Pediatrics, Division of Neonatology, Pediatric Intensive Care and Neuropediatrics, Medical University of Vienna, Waehringer Guertel 18-20, 1090 Vienna, Austria

^2^Comprehensive Center for Pediatrics, Medical University of Vienna, Waehringer Guertel 18-20, 1090 Vienna, Austria

^3^Department of Food Chemistry and Toxicology, University of Vienna, 1090 Vienna, Austria

^4^Joint Microbiome Facility of the Medical University of Vienna and the University of Vienna, Djerassiplatz 1, 1030 Vienna, Austria.

^5^Department of Microbiology and Ecosystem Science, Centre for Microbiology and Environmental Systems Science, University of Vienna, Djerassiplatz 1, 1030 Vienna, Austria.

^6^Department of Obstetrics and Gynecology, Division of Obstetrics and Feto-Maternal Medicine, Comprehensive Center for Pediatrics, Medical University of Vienna, 1090 Vienna, Austria.

^7^Exposome Austria, Research Infrastructure and National EIRENE Node, 1090 Vienna, Austria.

Correspondence to:

Dr. Lukas Wisgrill, PhD

Division of Neonatology, Paediatric Intensive Care & Neuropaediatrics
Department of Paediatrics and Adolescent Medicine
Comprehensive Center for Pediatrics
Medical University of Vienna
Währinger Gürtel 18-20
A-1090 Vienna
Austria

Email: lukas.wisgrill@meduniwien.ac.at
Telephone: +43 1 40400 29300
Fax: +43 1 40400 29290

**This file includes:**
Supplementary Materials and Methods
Figures S1 – S5
Table S1
Description for Supplementary File 1

**Supplementary Materials and Methods**

**Study population & Ethical approval**

For whole blood cytokine assays, samples were obtained from extremely low birth weight infants born before 28 weeks of gestation within the framework of the previously published *PreMiBraIn* cohort ^1,2^. Detailed information about inclusion and exclusion criteria, as well as prospective patient monitoring can be found in the respective publication ^1^. For monocyte-based assays, umbilical cord blood of seven premature infants born between 28 and 34 weeks of gestation (female n = 3) as well as six term-born infants (> 37 weeks of gestation, female n = 3) was collected aseptically into heparin tubes (Greiner Bio-One, Kremsmünster, Austria) directly from the placenta after caesarean section. Exclusion criteria for both cohorts were presence of maternal or neonatal infection at the day of delivery, congenital malformations or known genetic disorders of the child. To allow for comparison of immune training across human ontogeny, blood was also collected from the antecubital vein of nine healthy adults between 18 and 35 years of age (female n = 4). The study was approved by the ethics committee of the *Medical University of Vienna* (*EC-No. 1348/2017 & 2148/2017*).

**Isolation of monocytes and cell culture**

Peripheral blood mononuclear cells (PBMCs) were isolated using Ficoll-Paque density gradient centrifugation and resuspended in Dulbecco´s PBS (DPBS, GIBCO, Carlsbad, CA, USA). Subsequently, CD14^+^ cells were isolated using magnetic bead separation (anti-CD14 MicroBeads, Miltenyi Biotec, Bergisch-Gladbach, Germany). Afterwards, monocytes were transferred into a flat-bottom 96-well plate (Greiner Bio-One, Kremsmünster, Austria) at a density of 500.000 cells/mL and cultured in 200 µl RPMI-1640 medium (GIBCO, Carlsbad, CA, USA) supplemented with 10% human AB-serum (SeraCare Life Sciences Inc., Milford, MA, USA)^3^. Cells were kept in the presence or absence of 5 µg/mL β-Glucan (Invivogen, San Diego, CA, USA) for 24h at 37°C and 95% humidity. Unless indicated otherwise, samples were then centrifuged at 300g for 10 minutes, the supernatant was discarded, and monocytes rested in RPMI-medium until further experiments. Importantly, under these conditions, monocytes are known to differentiate into macrophages. However, because the β-Glucan training is initially conducted on monocytes whereas most downstream assays are performed on monocyte-derived macrophages, we collectively refer to cells as “monocytes” and “trained monocytes”, or directly state the treatment conditions of the cells within the full text.

**Measurement of monocyte cytokine responses**

To dissect cytokine responses in term and adult monocytes after β-Glucan training, β-Glucan-treated (6 days resting phase) and untreated cells were stimulated for 24h with 10 ng/mL ultrapure LPS (Invivogen, San Diego, CA, USA), 10 µg/mL Pam3-Cys (Invivogen, San Diego, CA, USA), or allowed to rest in fresh RPMI. Afterwards, samples were centrifuged, and the supernatants stored at -80°C. Cytokines were quantified from supernatant using the Human Custom ProcartaPlex 8-plex panel (Invitrogen, Waltham, MA, USA) with IFN-γ, IL-1β, IL-6, IL-8, IL-10, IL-12, IL-17A, and TNF-α as target analytes, according to manufacturer’s instructions.

**Measurement of whole-blood cytokine responses in preterm infants**

Heparinized whole blood was sampled from premature infants on day of life three, seven, 28 as well as at a corrected gestational age of 32 weeks and at term-corrected age. 25 µl of whole blood was transferred into a 96-well plate containing 250 µl AIM V medium (Thermo Fisher Scientific, Waltham, MA, USA). Samples were then stimulated for 24h with heat-killed *Escherichia coli* (HKEC, 1 x 10^8^, Invivogen, San Diego, CA, USA), *heat-killed Staphylococcus aureus* (HKSA, 1 x 10^8^, Invivogen, San Diego, CA, USA), or heat-killed *Candida albicans* (HKCA, 1 x 10^7^, Invivogen, San Diego, CA, USA), centrifuged, and the supernatants retrieved. Concentrations of IL-1β, IL-6, IL-8, IL-12 and TNF-α were quantified using the Human Custom ProcartaPlex panel (Invitrogen, Waltham, MA, USA), according to manufacturer’s instructions. To analyse the influence of inflammation/in-utero pathogen exposure on subsequent immune responses to heterologous pathogens in preterm infants we extracted laboratory information from routine measurements. We then identified patients who presented with elevated inflammatory markers (CRP > 2.2 mg dl^-1^ or IL-6 > 1000 pg ml^-1^) within three days of birth and patients who did not show inflammation after birth (CRP < 0.5 mg dl^-1^ and IL-6 < 150 pg ml^-1^) and compared cytokine responses to heterologous stimulation.

**Metabolomic profiling of monocytes by LC-HRMS**

For the analysis a Vanquish ultra-high-pressure liquid chromatography (UHPLC) system coupled to a QExactive HF quadrupole-Orbitrap mass spectrometer via an electrospray ionization (ESI) interface was used. A reverse phase (RP) column (Waters Acquity HSS T3; 1.8 μm, 100 x 2.1 mm) and a hydrophilic interaction liquid chromatography column (HILIC, SeQuant ZIC-pHILIC; 5 μm, 150 x 2.1 mm) with an injection volume of 3 μL, a flow rate of 0.3 mL/min and a column compartment temperature of 40°C were employed. The HILIC chromatography utilized H_2_O and acetonitrile (ACN) with 10 mM NH_4_HCO_3_ (90:10, v:v, pH 9.2, eluent A) and ACN (eluent B) for the following gradient: 0-6 min linear decrease from 75% B to 45% B, 6-7 min 45% B to 30% B, 7-10 min stay at 30% B, 10-10.1 min increase from 30% B to 75% B and was maintained constant at 75% B for 5 min. The RP chromatography utilized H_2_O with 0.1% formic acid (eluent A) and MeOH with 0.1% formic acid (eluent B) for the following gradient: 0-0.5 min hold at 5% B, 0.5-11.5 min linear increase to 95% B, 11.5-15.5 constant at 95% B, 15.5-15.6 decrease from 95% B to 5% B, 15.6-17 min stay at 5% B. Measurements were conducted in full-scan mode employing polarity switching and the following settings: sheath gas, 48 au; auxiliary gas, 15 au; sweep gas flow, 2 au; capillary voltage, 3.5 kV (positive), 2.8 kV (negative); capillary temperature, 300°C; auxiliary gas heater, 350°C; scan range m/z 62 to 900; resolution of 60.000; automatic gain control of 1 x 10^6^ and a maximum injection time of 200 ms. A pooled quality control, consisting of aliquots of all experimental samples, was prepared and injected at the beginning, the end and after every five consecutive experimental samples. A mixture of reference standards was injected at the beginning and the end of any sequence and used for metabolite identification. The software *Skyline (v. 20.2)* ^4^ was used to process the resulting *.raw files* and to perform a suspect screening for the metabolites contained in the reference standard mixture.

**RNA-extraction, paired-end sequencing and gene alignment**

For RNA-extraction, 1 x 10^6^ monocytes were cultured in alignment with the aforementioned conditions. After 24h of stimulation with 5 µg/ml β-Glucan, cells were allowed to rest in fresh medium for 6 days, detached using Accutase and washed once in PBS. Afterwards, RNA was extracted using the RNeasy Mini Kit (Qiagen, Hilden, Germany) according to manufacturer’s instructions. Sequencing libraries for polyA-enriched RNA isolated from the samples were prepared at the *Core Facility Genomics, Medical University of Vienna*. Libraries were quality controlled on a Bioanalyzer 2100 (Agilent, Santa Clara, CA, USA) using a high sensitivity DNA kit for correct insert size and quantified with the Qubit dsDNA HS Assay (Invitrogen, Waltham, MA, USA). Pooled libraries were sequenced on a NextSeq500 instrument (Illumina, San Diego, CA, USA) in 2 x 75bp paired-end sequencing mode. Approximately 30 x 10^6^ read pairs per sample were generated. Data were quality checked using *FastQC* ^5^ and cumulative reports generated with *multiQC* ^6^. Read lengths above 73bp were hard trimmed using *trimmomatic* ^7^ and the quality control was repeated. FastQ-files were aligned to the human reference genome version *GRCh38* available from *GENCODE v.45* ^8^ using *STAR v. 2.7.10a* ^9^. Genome indices were regenerated from splice junctions obtained during the first pass after filtering entries with less than two reads, non-canonical junctions, and already annotated junctions. Two pass mapping was then performed using newly assembled genome indices. To obtain count-matrices, the packages *Rsamtools* ^10^ and *GenomicAlignments* ^11^ were used according to a previously published workflow ^12^.

**Differential gene expression analysis**

Differentially expressed genes (DEG) were identified using the *limma* framework ^13^. Briefly, raw counts were log-transformed, cpm-transformed, and *TMM* used to account for between-sample variability. In alignment with current analysis strategies for complex nested designs, the *duplicate correlation* function was used to account for sample pairing and iterated twice. Consequently, contrasts were defined, and a linear model was fitted. After application of Benjamini-Hochberg correction (BH), genes with an adjusted p-value (adj. p-value) threshold below 0.05 and a log2-fold change (Log_2_FC) higher than 0.58 or lower than -0.58 were identified as differentially expressed. Results were visualized using the packages *ggplot2* ^14^, *VennDiagram* ^15^, *GoPlot* ^16^, and *ComplexHeatmap* ^17^.

**Overrepresentation analysis, gene set enrichment/variation analysis**

For overrepresentation analysis (ORA), DEG with an adj. p-value below 0.1 and a Log_2_FC higher than 0.58 or lower than -0.58 were selected. After manual adjustment of background genes, ORA was performed using the *enrichGO* function implemented in the *clusterProfiler* framework with default settings ^18^. Data were visualised using the *enrichplot* package ^19^. For gene set enrichment analysis (GSEA), Log_2_FC-ranked gene lists of respective contrasts were retrieved, and enrichment analysis performed using the *gseGO* function. Molecular function was selected as reference *GO-ontology* and gene set sizes adjusted manually. Afterwards, five significantly enriched and biologically relevant pathways were retrieved according to their gene ratio and visualised. Finally, the *heatplot* function was used to depict changes in the expression of genes with a Log_2_FC higher than 0.58 and lower than -0.58 (term vs. adult) and higher than 1 or lower than -1 (other comparisons) that were implicated in the activity of a pathway of a comparison of interest. Gene set variation analysis (GSVA) was performed using the GSVA package. Briefly, expression data were variance stabilized, human hallmark genes retrieved from the Molecular Signatures Database (MSigDB) and gene set variation analysis performed on the samples employing a minimum and maximum gene set size of 15 and 500, respectively. Significance was tested using the limma framework, as mentioned above.

**Analysis of lncRNA expression and impact on cellular pathways**

To analyse differences in lncRNA expression, the processed count matrix was filtered to only include lncRNAs that had previously been annotated in the *ENSEMBL* reference-database *“AH113665”*. Differentially expressed lncRNAs were then selected and depicted using *factoextra* ^20^ and *ComplexHeatmap* ^17^. Moreover, to convey biological importance to differentially expressed lncRNAs, pathway analysis was performed using the *lncPath* framework ^21^. Differentially expressed lncRNAs were assigned to their respective comparisons, mapped to a published coding-non-coding gene correlation network and propagation scores reflecting the influence of lncRNAs on respective target genes computed. Protein-coding target genes were then ranked according to this score, subjected to enrichment analysis using the *KEGG* database ^22^ and a permutation test with 10.000 repetitions performed. Subsequently, the five biologically most relevant pathways with an FDR < 0.05 were selected and depicted using a dotplot.

**Weighted gene co-expression network analysis (WGCNA)**

For *WGCNA* ^23^, a count matrix was initialized as a *DESeq2-object* ^24^, vsd-transformed and genes with low read counts removed. Afterwards, the interquartile range (IQR) of each *ENTREZ*-annotated gene was calculated across samples and the top 10.000 genes with the highest IQR selected as input for network construction. A signed network was constructed using the *blockwiseModules* function at a power of 12. A dissimilarity matrix was modelled and plotted to depict co-expression patterns. Eigengenes of respective gene modules were calculated, correlated to traits of interest, and visualised in a heatmap. Additionally, eigengenes of trait-correlated modules were extracted and their expression depicted over the course of maturation. To ascribe biological function to identified gene modules, ORA was performed using GO-terms for biological functions as ontology ^25^. For this exploratory analysis, biologically meaningful pathways with an adj. p-value below 0.1 were selected and visualized. Module membership was calculated for all genes deriving from modules of interest, genes ordered according to membership, and the expression of hub-genes depicted using boxplots.

**Co-expression network analysis using hCoCena**

Highly variable genes were selected according to hCoCena recommendations ^26^. Pearson correlations were calculated between genes, and a correlation cutoff of 0.82 was applied for network construction. An undirected integrated network was generated and clustered using the Leiden algorithm. Module scores, cluster heatmaps, and integrated network visualizations were generated using implemented functions/frameworks. Functional enrichment analysis was performed for all modules using Hallmark gene sets and p-values were corrected using Benjamini-Hochberg adjustment.

**Transcription factor enrichment analysis and functional network-based reverse mapping**

To ascertain differences in transcriptional regulation between conditions, a regulator-target network was constructed. For this, genes were mapped to the transcription factor-target gene network *CollecTRI* using the *decoupleR* framework ^27^. Regulator pathway activities were then calculated using t-values of respective comparisons. Afterwards, scores were corrected via multiplication with an empirical p-value deriving from a permutation test with 10.000 iterations. The ten most differentially positively and negatively enriched transcription factors were visualized. To cross-validate findings, transcription factors were reverse-mapped to their gene targets using the *TF-link* database ^28^. Obtained target genes were then subsetted according to differential expression and an ORA was performed to assess any biological context. Finally, because transcription factors tended to influence multiple pathways relevant to a condition of interest, their pathway activity was evaluated and depicted as a dotplot.

**Statistical analysis**

For the longitudinal comparison of cytokine responses between preterm infants who presented with in-utero pathogen exposure and non-inflamed controls, linear mixed models with the formula *log(cytokine) ~ group * timepoint + gestational age + (1 | patient)* were created. Further, estimated marginal means as well as 95% confidence intervals were calculated from the models for each respective timepoint, pairwise comparisons were performed and values were Bonferroni corrected to account for multiple testing. Regarding the comparative analysis of cytokine and metabolic alterations in response to immune training or LPS/ Pam3-Cys stimulation across ontogeny, linear models were created and adult responses set as reference for subsequent testing. Raw p-values were extracted from the model coefficients and Bonferroni corrected for multiple testing. Supplementary metabolomics data were analyzed using a two-way repeated measures ANOVA and significant main effects were explored using pairwise t-tests with Benjamini-Hochberg correction.

**Figure S1. Absolute concentrations of cytokines across ontogeny, stimulation and training**

***(A)*** *Absolute concentrations of cytokines measured from the supernatant of untrained monocytes derived from adults (green), term infants (blue) and preterm infants (red) stimulated with 10 ng/mL LPS for 24h after 6d of resting in RPMI.* ***(B)*** *Absolute concentrations of cytokines measured from the supernatant of untrained monocytes derived from adults (green), term infants (blue) and preterm infants (red) stimulated with 10 µg/mL Pam3-Cys for 24h after 6d of resting in RPMI.* ***(C)*** *Absolute concentrations of cytokines measured from the supernatant of monocytes trained with 5 µg/mL β-Glucan or untrained control monocytes, derived from adults (green), term infants (blue) and preterm infants (red) upon stimulation with 10 µg/mL Pam3-Cys for 24h after a 6d washout period in RPMI had concluded.*

**Figure S2. Additionally quantified metabolites**

*Relative abundances (AUC) of metabolites not shown in Figure 2 are depicted for β-Glucan-treated (violet) and untreated (beige) monocytes derived from adults (green, n = 4), term-born infants (blue, n = 5), and preterm infants (red, n = 3).*

**Figure S3. Paired analysis of metabolomics data**

***(A)*** *Conventional principal component analysis of all samples included in Figure 2A. Color coding represents sample pairing (two samples per donor) to depict interindividual differences as the main driver of separation along the first two principal components. Circles (adults, n = 4), rectangles (term infants, n = 5), and triangles (preterm infants, n = 3) indicate sample ontogeny.* ***(B)*** *Relative abundances (AUC) of metabolites shown in Figure 2B, depicted as boxplots with lines connecting paired data points across treatment conditions for cells derived from adults (green, n = 4), term-born infants (blue, n = 5), and preterm infants (red, n = 3). Data were analyzed using a two-way repeated-measures ANOVA, and significant main effects were explored using pairwise t-tests with BH correction. •p < 0.1; *p < 0.05; **p < 0.01; ***p < 0.001*

**Figure S4. GSVA analysis across treatment and maturation**

***(A)*** *Heatmap (hierarchical clustering) depicting the change in GSVA enrichment scores (delta GSVA) of hallmark gene sets after treatment with β-glucan for 24h and resting of cells for 6 days for adults (green, n = 6), term infants (blue, n = 5), and preterm infants (red, n = 3).* ***(B)*** *GSVA scores of selected pathways across treatment (mock = beige, β-glucan = violet) and ontogeny. Data were analyzed using the limma framework with BH correction. •p < 0.1; *p < 0.05; **p < 0.01; ***p < 0.001*

**Figure S5. Co-expression network analysis using hCoCena**

***(A)*** *Heatmap displaying the mean group fold change (GFC) of co-expression modules (M1–M3) across treatment (β-Glucan = violet, mock = beige) and ontogeny (adult = green, term = blue, preterm = red).* ***(B)*** *Co-expression network with nodes representing genes and edges representing co-expression relationships. Nodes are colored by module membership (M1 = blue, M2 = yellow, M3 = green).* ***(C)*** *Module-specific (M1–M3) functional enrichment using Hallmark gene sets from MSigDB with dot size corresponding to enrichment significance.*

| **Compound** | **CAS number** | **Identification Confidence Level** |
| --- | --- | --- |
| 3-Methyl-2-oxovaleric acid | 1460-34-0 | 1 |
| 4-Hydroxy-proline | 6912-67-0 | 1 |
| 5'-Deoxy-5'-Methylthioadenosine | 2457-80-9 | 1 |
| Adenine | 73-24-5 | 1 |
| Adenosine | 58-61-7 | 1 |
| Adenosine 3',5'-cyclic monophosphate | 60-92-4 | 1 |
| Alanine | 302-72-7 | 1 |
| alpha-Ketoglutarate | 328-50-7 | 1 |
| Arginine | 74-79-3 | 1 |
| Asparagine | 70-47-3 | 1 |
| Aspartic acid | 56-84-8 | 1 |
| Betaine | 107-43-7 | 1 |
| Carnitine | 406-76-8 | 1 |
| cis-Aconitate | 585-84-2 | 1 |
| Cysteic acid | 13100-82-8 | 1 |
| Dihydroxyacetone phosphate | 57-04-5 | 1 |
| Flavin adenine dinucleotide | 146-14-5 | 1 |
| Fumarate | 142-42-7 | 1 |
| Glutamate | 56-86-0 | 1 |
| Glutamine | 56-85-9 | 1 |
| Glycine | 56-40-6 | 1 |
| Glyoxylic acid | 298-12-4 | 1 |
| Guanidineacetic acid | 352-97-6 | 1 |
| Histidine | 71-00-1 | 1 |
| Homoserine | 1927-25-9 | 1 |
| Hydroxyglutaric acid | 2889-31-8 | 1 |
| Isoleucine | 443-79-8 | 1 |
| Ketoisovalerate | 759-05-7 | 1 |
| Lactate | 113-21-3 | 1 |
| L-Citrulline | 372-75-8 | 1 |
| Leucine | 61-90-5 | 1 |
| L-Ornithine | 70-26-8 | 1 |
| Lysine | 56-87-1 | 1 |
| Malic acid (Malate) | 320-77-4 | 1 |
| Mannitol | 69-65-8 | 1 |
| Methionine | 63-68-3 | 1 |
| N-Acetyl-L-aspartic acid | 997-55-7 | 1 |
| Nicotinamide | 98-92-0 | 1 |
| Phenylalanine | 63-91-2 | 1 |
| Proline | 4305-67-3 | 1 |
| Propionyl-L-carnitine | 17298-37-2 | 1 |
| Pseudouridine | 1445-07-4 | 1 |
| Pyruvate | 57-60-3 | 1 |
| Serine | 56-45-1 | 1 |
| Spermidine | 124-20-9 | 1 |
| Succinate | 56-14-4 | 1 |
| Threonine | 72-19-5 | 1 |
| Tryptophan | 73-22-3 | 1 |
| Tyrosine | 60-18-4 | 1 |
| Uridine | 58-96-8 | 1 |
| Valine | 72-18-4 | 1 |
| Xanthine | 69-89-6 | 1 |

**Table S1. Metabolites quantified by LC-HRMS, with CAS registry numbers and identification confidence levels.**

**Supplementary File 1. Differentially expressed genes for all contrasts and WGCNA module MEM14 gene membership (provided as a separate Excel file).**

*Differentially expressed genes were identified using the limma framework, as explained in the Materials and Methods section. The file contains one worksheet per contrast (9 contrasts in total). For each contrast, all genes that reached statistical significance at an adj. p-value < 0.05 are listed. No cut-off for effect size has been applied. Adult_Effect, Term_Effect and Preterm_Effect refer to the effect of β-glucan training (trained versus untrained cells) within adult, term, and preterm monocytes, respectively. Gluc_AdTe, Gluc_AdPre, and Gluc_PreTe, report between-group contrasts among β-glucan-trained cells (Ad = Adult, Te = Term, Pre = Preterm). MOCK_AdTe, MOCK_AdPre, and MOCK_PreTe, display the corresponding contrasts among untrained cells (Ad = Adult, Te = Term, Pre = Preterm). The final worksheet, Module_M14, lists all genes assigned to module 14 by WGCNA. Module M14 was the only module directly associated with β-glucan training and is included for transparency, as no plausible biological function could be inferred using overrepresentation analysis.*

**References**

1.	Seki D, Mayer M, Hausmann B, et al. Aberrant gut-microbiota-immune-brain axis development in premature neonates with brain damage. *Cell Host & Microbe*. 2021;29(10):1558-1572.e6.

2.	Pristner M, Wasinger D, Seki D, et al. Neuroactive metabolites and bile acids are altered in extremely premature infants with brain injury. *Cell Reports Medicine*. 2024;5(4):101480.

3.	Namakula R, De Bree LCJ, A. Tvedt TH, Netea MG, Cose S, Hanevik K. Monocytes from neonates and adults have a similar capacity to adapt their cytokine production after previous exposure to BCG and β-glucan. Izzo AA, ed. *PLoS ONE*. 2020;15(2):e0229287.

4.	Adams KJ, Pratt B, Bose N, et al. Skyline for Small Molecules: A Unifying Software Package for Quantitative Metabolomics. *J Proteome Res*. 2020;19(4):1447-1458.

5.	Andrews S. FastQC: a quality control tool for high throughput sequence data. 2010.

6.	Ewels P, Magnusson M, Lundin S, Käller M. MultiQC: summarize analysis results for multiple tools and samples in a single report. *Bioinformatics*. 2016;32(19):3047-3048.

7.	Bolger AM, Lohse M, Usadel B. Trimmomatic: a flexible trimmer for Illumina sequence data. *Bioinformatics*. 2014;30(15):2114-2120.

8.	Frankish A, Diekhans M, Ferreira AM, et al. GENCODE reference annotation for the human and mouse genomes. *Nucleic Acids Research*. 2019;47(D1):D766-D773.

9.	Dobin A, Davis CA, Schlesinger F, et al. STAR: ultrafast universal RNA-seq aligner. *Bioinformatics*. 2013;29(1):15-21.

10.	Martin Morgan HP. Rsamtools. Published online 2017.

11.	Hervé Pagès VO. GenomicAlignments. Published online 2017.

12.	Love MI, Anders S, Kim V, Huber W. RNA-Seq workflow: gene-level exploratory analysis and differential expression. *F1000Res*. 2015;4:1070.

13.	Gordon Smyth A. limma. Published online 2017.

14.	Wickham H. *Ggplot2: Elegant Graphics for Data Analysis*. Second edition. Springer; 2016.

15.	Chen H, Boutros PC. VennDiagram: a package for the generation of highly-customizable Venn and Euler diagrams in R. *BMC Bioinformatics*. 2011;12(1):35.

16.	Walter W, Sánchez-Cabo F, Ricote M. GOplot: an R package for visually combining expression data with functional analysis. *Bioinformatics*. 2015;31(17):2912-2914.

17.	Zuguang Gu. ComplexHeatmap. Published online 2017.

18.	Yu G, Wang LG, Han Y, He QY. clusterProfiler: an R Package for Comparing Biological Themes Among Gene Clusters. *OMICS: A Journal of Integrative Biology*. 2012;16(5):284-287.

19.	Guangchuang Yu. enrichplot. Published online 2018.

20.	Alboukadel K, Mundt F. Extract and Visualize the Results of Multivariate Data Analyses [R package factoextra version 1.0.7]. Published online 2020.

21.	Han J, Liu S, Sun Z, et al. LncRNAs2Pathways: Identifying the pathways influenced by a set of lncRNAs of interest based on a global network propagation method. *Sci Rep*. 2017;7(1):46566.

22.	Kanehisa M. KEGG: Kyoto Encyclopedia of Genes and Genomes. *Nucleic Acids Research*. 2000;28(1):27-30.

23.	Langfelder P, Horvath S. WGCNA: an R package for weighted correlation network analysis. *BMC Bioinformatics*. 2008;9(1):559.

24.	Simon Anders Michael Love. DESeq2. Published online 2017.

25.	Subramanian A, Tamayo P, Mootha VK, et al. Gene set enrichment analysis: A knowledge-based approach for interpreting genome-wide expression profiles. *Proc Natl Acad Sci USA*. 2005;102(43):15545-15550.

26.	Holsten L, Dahm K, Oestreich M, Becker M, Ulas T. hCoCena: A toolbox for network-based co-expression analysis and horizontal integration of transcriptomic datasets. *STAR Protoc*. 2024;5(1):102922.

27.	Badia-i-Mompel P, Vélez Santiago J, Braunger J, et al. decoupleR: ensemble of computational methods to infer biological activities from omics data. Kuijjer ML, ed. *Bioinformatics Advances*. 2022;2(1):vbac016.

28.	Liska O, Bohár B, Hidas A, et al. TFLink: an integrated gateway to access transcription factor–target gene interactions for multiple species. *Database*. 2022;2022:baac083.
